# Antihypertensive Treatment and Central Arterial Hemodynamics: A Meta-Analysis of Randomized Controlled Trials

**DOI:** 10.3389/fphys.2021.762586

**Published:** 2021-11-24

**Authors:** Yi-Bang Cheng, Jia-Hui Xia, Yan Li, Ji-Guang Wang

**Affiliations:** Shanghai Key Laboratory of Hypertension, Department of Cardiovascular Medicine, Centre for Epidemiological Studies and Clinical Trials, National Research Centre for Translational Medicine, Ruijin Hospital, Shanghai Institute of Hypertension, Shanghai Jiao Tong University School of Medicine, Shanghai, China

**Keywords:** antihypertensive treatment, central blood pressure, augmentation index, randomized controlled trial, drug

## Abstract

**Background:** Antihypertensive treatment may have different effects on central arterial hemodynamics. The extent of the difference in effects between various antihypertensive drugs remains undefined.

**Methods:** We conducted a systematic review and meta-analysis of randomized controlled trials that explored the effects of antihypertensive agents on both central and peripheral systolic blood pressure (SBP) and pulse pressure (PP) or central augmentation index, with a special focus on the comparison between newer [renin-angiotensin-aldosterone system (RAS) inhibitors and calcium-channel blockers (CCBs)] and older antihypertensive agents (diuretics and β- and α-blockers).

**Results:** In total, 20 studies (*n* = 2,498) were included. Compared with diuretics (10 studies), β-blockers (16 studies), or an α-blocker (1 study), RAS inhibitors (21 studies), and CCBs (6 studies) more efficaciously (*P* < 0.001) reduced both central and peripheral SBP by a weighted mean difference of −5.63 (−6.50 to −4.76 mmHg) and −1.97 mmHg (−2.99 to −0.95 mmHg), respectively. Compared with older agents, the newer agents also more efficaciously (*P* < 0.001) reduced central PP (−3.27 mmHg; −4.95 to −1.59 mmHg), augmentation index (−6.11%; −7.94 to −4.29) and augmentation (−3.35 mmHg; −5.28 to –1.42 mmHg) but not peripheral PP (*p* ≥ 0.09). Accordingly, the newer agents reduced central-to-peripheral PP amplification significantly less than the older agents (0.11 mmHg; 0.05 to 0.17 mmHg; *P* < 0.001).

**Conclusion:** Newer agents, such as RAS inhibitors and CCBs, were significantly more efficacious than older agents in their effects on central hemodynamics.

## Introduction

When the blood flows from the central large elastic aorta to the peripheral smaller muscular arteries, systolic blood pressure (SBP) increases, without significant changes in diastolic blood pressure and mean arterial pressure, resulting in widened pulse pressure (PP). Although brachial blood pressure highly correlates with central blood pressure, substantial individual discrepancies between the central and peripheral blood pressure exist. Several studies have shown that the association between target-organ damage and SBP and PP is stronger for the central arteries than the brachial arteries (Kollias et al., 2016). Indeed, in a meta-analysis of 11 studies that included 5,648 subjects followed up for 3.8 years, central PP showed borderline superiority to brachial PP in the prediction of cardiovascular events (Vlachopoulos et al., 2010). Similar results were obtained in at least two recent systematic reviews and meta-analyses (Li et al., 2019; Vieceli et al., 2021).

Previous studies have shown that various classes of antihypertensive drugs may have different treatment effects between the central and peripheral arterial sites and that the newer antihypertensive agents, such as renin-angiotensin-aldosterone (RAS) inhibitors and calcium-channel blockers (CCBs), might be more efficacious than the older ones, such as diuretics, β-blockers, and α-blockers in the effect on central hemodynamics. The Conduit Artery Function Evaluation (CAFE) study, a substudy of the Anglo-Scandinavian Cardiac Outcomes Trial (ASCOT), showed that treatment with amlodipine/perindopril was more efficacious than with atenolol/bendroflume thiazide in reducing central SBP and PP by 4.3 and 3.0 mmHg, respectively, despite similar reductions in the brachial arteries (Williams et al., 2006). The cardiovascular benefits of treatment with amlodipine and perindopril observed in ASCOT (Dahlöf et al., 2005) might have been resulted at least in part from the lowering of central blood pressure, although other hemodynamic effects, such as reduced blood pressure variability, might have also played a part (Rothwell et al., 2010). In fact, there is a growing interest in central blood pressure as a target of treatment in hypertension.

In this comparative meta-analysis of randomized controlled trials, we investigated the effects of the newer agents vs. older antihypertensive agents on various central hemodynamic measurements.

## Materials and Methods

### Search Strategy and Selection Criteria

Our meta-analysis strictly followed the recommendations of the *Preferred Reporting Items for Systematic Reviews and Meta-Analysis: The PRISMA Statement* (Moher et al., 2009). A total of 5,158 abstracts and full-text articles were retrieved systematically from electronic databases (PubMed, Embase, and Cochrane Central Register of Controlled Trials) and searched manually on September 15, 2020. The search key terms included “central pressure,” “aortic pressure,” “carotid pressure,” “pulse amplification,” “central-to-peripheral pulse pressure ratio,” “augmentation index,” “antihypertensive drug,” “antihypertensive treatment,” and “antihypertensive agent.” We limited our search to studies published in peer-reviewed journals in English. We checked the reference lists of review and original articles identified by the electronic search to find other potentially eligible studies.

The selection criteria for the inclusion of clinical trials in this meta-analysis were as follows: parallel-group randomized actively controlled trials in humans, the duration of treatment was no less than 4 weeks, and peripheral and central SBP or augmentation index after intervention were reported in a published article. Studies were excluded if the intervention was not an antihypertensive drug, or if the comparison was within the same drug class, between two newer (RAS inhibitors and CCBs) or older agents (diuretics and β- or α-blockers) or with a combination antihypertensive therapy.

### Data Extraction and Quality Assessment

Data extraction was performed using predefined data fields. Variables included author name, year of publication, study design, study population, number of patients, study intervention, duration of treatment and specifications of the blood pressure measuring device and other methods for blood pressure measurement, arterial sites, and the algorithm for augmentation index estimation. Baseline and post-intervention mean values of central and peripheral hemodynamic measurements for the experimental and control groups were extracted with standard deviation (SD), standard error of mean (SEM), or 95% confidence intervals (CIs) separately.

Central hemodynamic measurements included central systolic and diastolic blood pressure (mmHg), central PP (mmHg), augmentation pressure (mmHg), and augmentation index (%). Central augmentation pressure was the absolute difference between the second peak (P2) and the first peak (P1) of the central blood pressure wave. The central augmentation index was calculated either as the ratio of the P2 to the P1, or as augmentation pressure (P2−P1) divided by PP, expressed in percent. Peripheral measurements were brachial SBP and diastolic blood pressure and PP. Central-to-peripheral PP amplification was calculated as the ratio of the central PP to the peripheral PP.

When multiple usable groups were available within an individual study, the data were counted as another study in the meta-analysis. Methodological quality was assessed using the Jadad scores (Jadad et al., 1996). Study selection, quality assessment, and data extraction were performed independently by two investigators (Y-BC and J-HX) in an unblinded standardized manner. Disagreements were resolved by negotiation or consensus with a third authoritative investigator (J-GW).

### Data Analysis

For each comparison within each trial, we calculated the absolute differences between the experimental and control groups. If significant between-group differences in any outcome measure were reported at baseline, we calculated the absolute difference in the mean changes over time. The pooled effect for each grouping of trials was derived from the point estimate for each separate trial weighted by the inverse of the variance (1/SE^2^). Heterogeneity of effect sizes was tested across trials using the χ^2^ test. If trials were homogeneous (*p* < 0.10), a fixed-effects model was used to calculate pooled effect sizes. Otherwise, a random-effects model was applied to calculate overall differences. Net treatment effects on central pressure and augmentation index were determined by subtracting the mean change in the experimental group from the corresponding mean change in the control group. We performed all aforementioned computations and statistical analyses in Stata version 15 (Stata Corp LP, College Station, TX, United States). SEM was converted to SD [SE = SD/√(sample size)], and CIs were calculated [CI = mean difference ± (SEM × 1.96)], as appropriate.

Publication bias was assessed using the Egger’s statistic and visual inspection of funnel plots. Potential heterogeneity was further inspected by visual inspection of the data and by subgroup and sensitivity analyses. We performed subgroup analyses based on the classes of drugs, sensitivity analyses by limiting to studies with a Jadad score of ≥3, central blood pressure *via* the radial approach with the SphygmoCor device (AtCor Medical, Sydney, NSW, Australia), and a primary diagnosis of hypertension. All *p*-values were calculated from two-tailed tests of statistical significance with a type 1 error of 5%.

## Results

### Characteristics of the Studies

**FIGURE 1:**
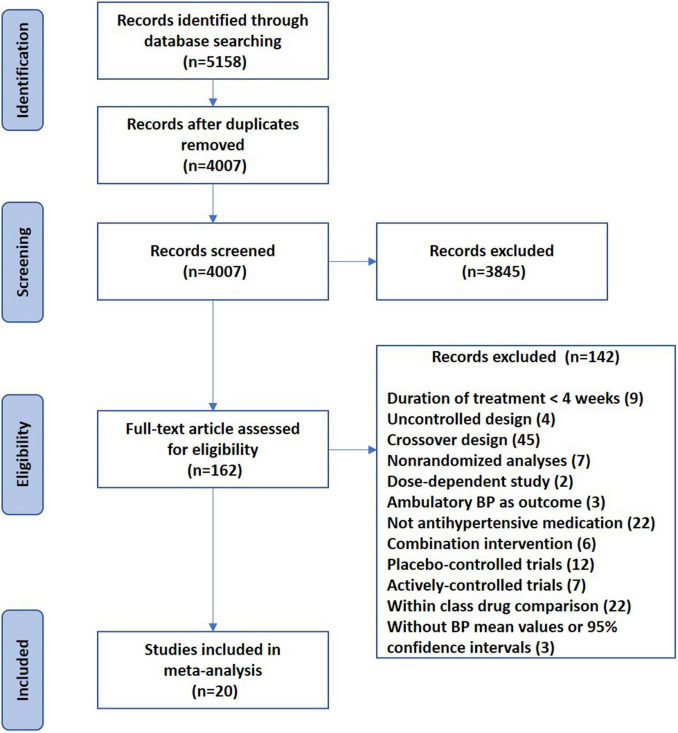
Flow diagram of the selection procedure for studies.

Figure 1 shows the flow diagram of the selection procedure for studies. The initial literature search retrieved 5,158 potentially eligible articles, and 4,007 records remained after removing duplicates. After having reviewed the title and abstract, 3,845 were excluded. Of the 162 full-text articles retrieved, 142 original articles were excluded for various reasons (Figure 1), leaving 20 eligible original articles in the analysis (Chen et al., 1995; Klingbeil et al., 2002; Ariff et al., 2006; Dart et al., 2007; Jiang et al., 2007; Schneider et al., 2008; Mackenzie et al., 2009; Matsui et al., 2009; Boutouyrie et al., 2010; Doi et al., 2010; Vitale et al., 2012; Kubota et al., 2013; Radchenko et al., 2013; Kim et al., 2014; Koumaras et al., 2014; Ghiadoni et al., 2017; Jekell et al., 2017; Miyoshi et al., 2017; Webster et al., 2017; Choi et al., 2018), comparing RAS inhibitors (*n* = 21) or CCBs (*n* = 6) with diuretics, β-blockers, or an α-blocker (Table 1).

**TABLE 1:** Trials of the renin-angiotensin-aldosterone system (RAS) inhibitors and calcium-channel blockers (CCBs) vs. diuretics, β-blockers, and α-blockers.

First author	Year	Blinding	Patients	No of patients	Treatment	Arterial site	Device	Algorithm*	Measurements	Follow-up	Jadad
**ACEIs vs. diuretics**											
Dart A. M.	2007	Open	HT	479	ACEI vs. diuretic	Carotid	Millar	–	cBP	4 y	3
Jiang X. J.	2007	Double	HT	101	Enalapril vs. indapamide	Radial	SphygmoCor	(P2−P1)/PP	cBP and AI	8 w	3
Mackenzie I. S.	2009	Double	HT	28	Perindopril vs. bendrofluazide	Radial	SphygmoCor	(P2−P1)/PP	cBP, AP, and AI	10 w	3

**ACEIs vs. β -blockers**											
Chen C. H.	1995	Double	HT	79	Fosinopril vs. atenolol	Carotid	Millar	(P2−P1)/PP	AI	8 w	2
Mackenzie I. S.	2009	Double	HT	32	Perindopril vs. atenolol	Radial	SphygmoCor	(P2−P1)/PP	cBP, AP, and AI	10 w	3
Koumaras C.	2014	Unknown	HT	37	Quinapril vs. atenolol	Radial	SphygmoCor	(P2−P1)/PP	cBP and AI	10 w	2
Koumaras C.	2014	Unknown	HT	37	Quinapril vs. nebivolol	Radial	SphygmoCor	(P2−P1)/PP	cBP and AI	10 w	2

**ACEIs vs. α -blockers**											
Jekell A.	2017	Double	HT	61	Doxazosin vs. ramipril	Radial	SphygmoCor	(P2−P1)/PP	cBP and AI	12 w	7

**Angiotensin-receptor blockers (ARBs) vs. diuretics**											
Klingbeil A. U.	2002	Double	HT	40	Valsartan vs. HCTZ	Radial	SphygmoCor	P2/P1	cBP, AP, and AI	6 w	4

**ARBs vs. β -blockers**											
Ariff B.	2006	Double	HT	88	Candesartan vs. atenolol	Carotid	Millar	–	cBP	52 w	3
Schneider M. P.	2008	Double	HT	156	Irbesartan vs. atenolol	Radial	SphygmoCor	(P2−P1)/PP	cBP, AP, and AI	18 m	3
Boutouyrie P.	2010	Open	HT	393	Valsartan vs. atenolol	Radial	SphygmoCor	(P2−P1)/PP	cBP and AI	24 w	5
Radchenko G. D.	2013	Open	HT	59	Losartan vs. bisoprolol	Radial	SphygmoCor	(P2−P1)/PP	cBP and AI	6 m	2
Choi M. H.	2018	Double	Ischemic stroke	70	Valsartan vs. atenolol	Radial	Omron	(P2−P1)/PP	cBP and AI	12 w	4
Choi M. H.	2018	Double	Ischemic stroke	70	Fimasartan vs. atenolol	Radial	Omron	(P2−P1)/PP	cBP and AI	12 w	4
Kim E. J.	2014	Open	HT	182	Losartan vs. carvedilol	Radial	Hanbyul Meditech	(P2−P1)/PP	cBP and AI	24 w	5
Vitale C.	2012	Double	HT	65	Irbesartan vs. nebivolol	Radial	SphygmoCor	(P2−P1)/PP	cBP and AI	8 w	5

**Renin inhibitors vs. diuretics**											
Kubota Y.	2013	Open	HT	30	Aliskiren vs. HCTZ	Radial	Omron	P2/P1	cBP and AI	12 w	2
Miyoshi T.	2017	Open	HT	97	Aliskiren vs. trichlormethiazide	Radial	Omron	P2/P1	cBP and AI	24 w	5

**Renin inhibitors vs. β -blockers**											
Koumaras C.	2014	Unknown	HT	35	Aliskiren vs. atenolol	Radial	SphygmoCor	(P2−P1)/PP	cBP and AI	10 w	2
Koumaras C.	2014	Unknown	HT	35	Aliskiren vs. nebivolol	Radial	SphygmoCor	(P2−P1)/PP	cBP and AI	10 w	2

**CCBs vs. diuretics**											
Mackenzie I. S.	2009	Double	HT	27	Lercanidipine vs. bendrofluazide	Radial	SphygmoCor	(P2−P1)/PP	cBP, AP, and AI	10 w	4
Matsui Y.	2009	Open	HT	207	Azelnidipine vs. HCTZ	Radial	SphygmoCor	(P2−P1)/PP	cBP, AP, and AI	24 w	3
Doi M.	2010	Open	HT	37	Azelnidipine vs. trichlormethiazide	Radial	Omron	P2/P1	cBP and AI	6 m	3
Ghiadoni L.	2017	Open	Metabolic syndrome	76	Lercanidipine vs. HCTZ	Radial	SphygmoCor	(P2−P1)/PP@HR75	cBP, AP, and AI	24 w	3

**CCBs vs. β -blockers**											
Mackenzie I. S.	2009	Double	HT	31	Lercanidipine vs. atenolol	Radial	SphygmoCor	(P2−P1)/PP	cBP, AP, and AI	10 w	3
Webster L. M.	2017	Open	HT in pregnancy	112	Nifedipine vs. labetalol	Brachial	Arteriograph	(P2−P1)/PP	cBP and AI	130 d	2

Studies are listed in the order of the year of publication per category. *Augmentation index was calculated either by the ratio of the second peak (P2) to the first peak (P1) of the central blood pressure wave or by augmentation pressure (P2−P1) divided by PP, expressed in percent. @HR75 indicated that the augmentation index was adjusted by heart rate at 75 bpm. d, days; w, weeks; m, months; y, years; HT, hypertension; HCTZ, hydrochlorothiazide; cBP, central blood pressure; AP, augmentation pressure; AI, augmentation index.

These 20 trials included a total of 2,498 participants. The mean age of the study participants ranged from 35.5 (Webster et al., 2017) to 71.6 years (Dart et al., 2007), the proportion of women from 21.0% (Ghiadoni et al., 2017) to 100% (Webster et al., 2017), and the mean follow-up time from 6 (Klingbeil et al., 2002) to 52 weeks (Ariff et al., 2006). The study design was double-blinded in 10 studies, open in 9 studies, and not reported in 1 study. Central hemodynamics was estimated non-invasively from radial, carotid, and brachial applanation tonometry in 16 studies, 3 studies, and 1 study, respectively. Radial tonometry was performed using the SphygmoCor device (*n* = 11), (Omron Healthcare, Kyoto, Japan) (*n* = 4), or (Hanbyul Meditech, Jeonju, Korea) (*n* = 1).

### Renin-Angiotensin-Aldosterone System Inhibitors and Calcium-Channel Blockers vs. Diuretics, β-Blockers, and α-Blockers

In total, we performed analyses in 20 trials with 1,250 and 1,248 participants in the treatment groups of newer and older antihypertensive drugs, respectively (Table 1). Newer antihypertensive agents consisted of an ACEI in eight trials (*n* = 854), an ARB in nine trials (*n* = 1,123), a renin inhibitor in four trials (*n* = 197), and a CCB in six trials (*n* = 490).

**FIGURE 2:**
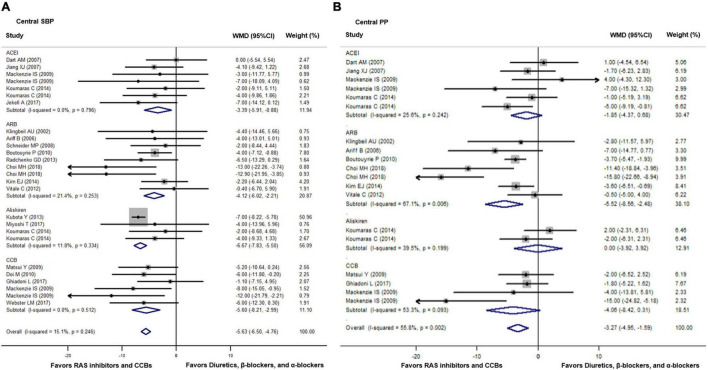
Effect of angiotensin-converting enzyme inhibitors (ACEIs), angiotensin-receptor blockers (ARBs), renin inhibitors, and calcium-channel blockers (CCBs) vs. diuretics, β-blockers, and α-blockers on central systolic blood pressure (SBP, **A**) and pulse pressure (PP, **B**). Weights are from the fixed **(A)** and random **(B)** effects analyses. RAS indicates renin-angiotensin-aldosterone system. Dots represent mean difference of each study. The size of the squares is proportional to the sample size. Horizontal lines represent the 95% confidence intervals (CI). Open diamonds represent the weighted mean difference (WMD) with 95% CI.

Compared with diuretics (*n* = 10), β-blockers (*n* = 16), or an α-blocker (*n* = 1), the weighted mean differences in the central SBP were statistically significant for ACEIs (*n* = 7) by −3.39 mmHg (−5.91 to −0.89, *p* = 0.008), for ARBs (*n* = 9) by −4.12 mmHg (−6.02 to −2.21, *p* < 0.001), for renin inhibitors (*n* = 4) by −6.67 mmHg (−7.83 to −5.50, *p* < 0.001), and for CCBs (*n* = 6) by −5.60 mmHg (−8.21 to −2.99, *p* < 0.001). No significant heterogeneity was noticed within all four classes of newer drugs (*p* ≥ 0.24). The overall weighted mean difference in the central SBP across all 20 trials was −5.63 mmHg (−6.50 to −4.76, *p* < 0.001; *I*^2^ = 15.1%, *P* for heterogeneity = 0.25). The weighted mean differences in central PP were statistically significant for ARBs (*n* = 7) by −5.52 mmHg (−8.56 to −2.48, *p* = 0.006) but not significant for ACEIs (*n* = 6), renin inhibitors (*n* = 2), or CCBs (*n* = 4, *p* ≥ 0.07). The overall weighted mean difference in central PP across 12 studies with data was −3.27 mmHg (−4.95 to −1.59, *p* < 0.001; *I*^2^ = 55.8%, *P* for heterogeneity = 0.002, Figure 2).

**FIGURE 3:**
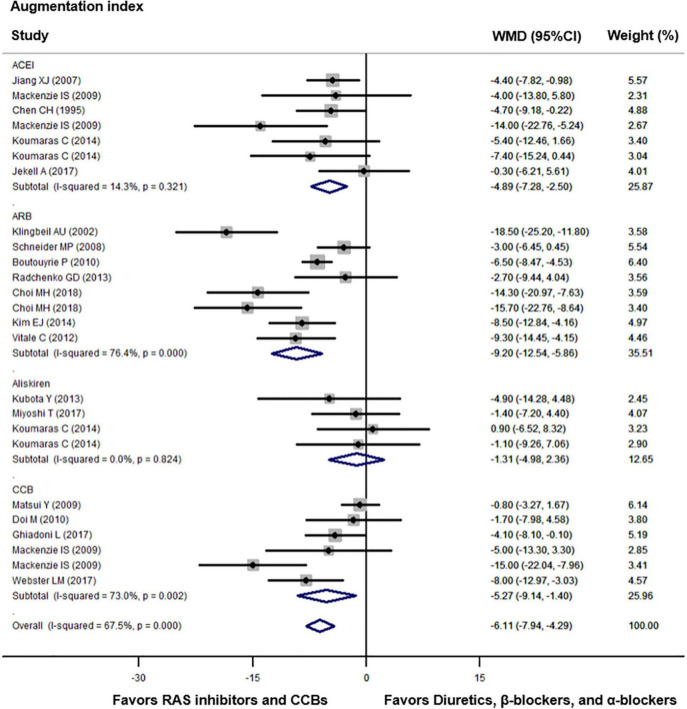
Effect of angiotensin-converting enzyme inhibitors (ACEIs), angiotensin-receptor blockers (ARBs), renin inhibitors, and calcium-channel blockers (CCBs) vs. diuretics, β-blockers, and α-blockers on central augmentation index. Weights are from the random-effects analysis. For further details, see legends in Figure 2.

**FIGURE 4:**
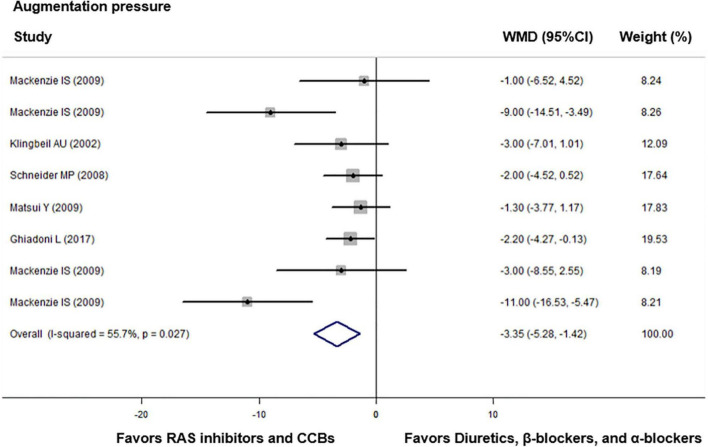
Effect of angiotensin-converting enzyme inhibitors (ACEIs), angiotensin-receptor blockers (ARBs), renin inhibitors, and calcium-channel blockers (CCBs) vs. diuretics, β-blockers, and α-blockers on central augmentation pressure. Weights are from the fixed-effects analysis. For further details, see legends in Figure 2.

Compared with diuretics (*n* = 9), β-blockers (*n* = 15), or an α-blocker (*n* = 1), the weighted mean differences in the central augmentation index were statistically significant for ACEIs (*n* = 7) by −4.89% (−7.28 to −2.50, *p* = 0.001), for ARBs (*n* = 8) by −9.20% (−12.54 to −5.86, *p* < 0.001), and for CCBs (*n* = 6) by −5.27% (−9.14 to −1.40, *p* = 0.008). The overall weighted mean difference in the central augmentation index across 18 studies with data was −6.11% (−7.94 to −4.29, *p* < 0.001; *I*^2^ = 67.5%, *P* for heterogeneity < 0.001, Figure 3). In addition, across five trials with data, the overall weighted mean difference in the central augmentation pressure was −3.35 mmHg (−5.28 to −1.42, *p* < 0.001; *I*^2^ = 55.7%, *P* for heterogeneity = 0.03, Figure 4).

With regard to peripheral measurements, the weighted mean difference was significant for SBP across 19 trials by −1.97 mmHg (−2.99 to −0.95, *p* < 0.001; *I*^2^ = 0.0%, *P* for heterogeneity = 0.67), but not for PP across 9 studies [−0.90 mmHg (−1.92 to 0.13 mmHg), *p* = 0.09; *I*^2^ = 25.8%, *P* for heterogeneity = 0.16, Supplementary Figure 1].

**FIGURE 5:**
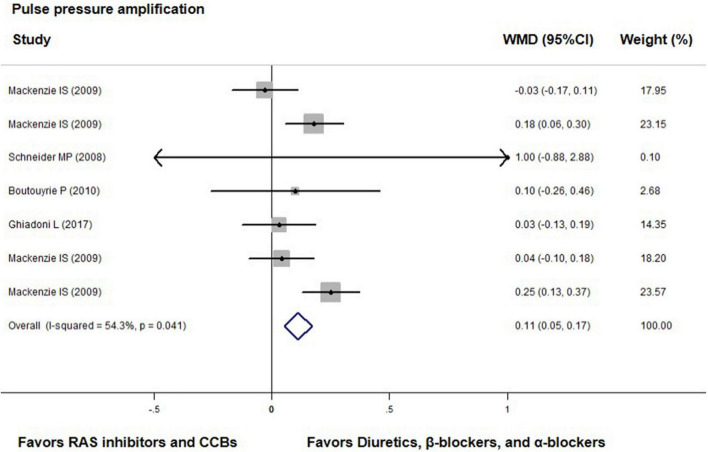
Effect of angiotensin-converting enzyme inhibitors (ACEIs), angiotensin-receptor blockers (ARBs), renin inhibitors, and calcium-channel blockers (CCBs) vs. diuretics, β-blockers, and α-blockers on central-to-peripheral pulse pressure amplification. Weights are from the fixed-effects analysis. For further details, see legends in Figure 2.

Compared with diuretics (*n* = 3) or β-blockers (*n* = 4), the overall weighted mean differences in central-to-peripheral PP amplification were significantly smaller across four trials with either ACEIs (*n* = 2), ARBs (*n* = 2), or CCBs (*n* = 3) by 0.11 mmHg (0.05 to 0.17, *p* < 0.001; *I*^2^ = 54.3%, *P* for heterogeneity = 0.041, Figure 5).

### Publication Bias and Sensitivity Analysis

**FIGURE 6:**
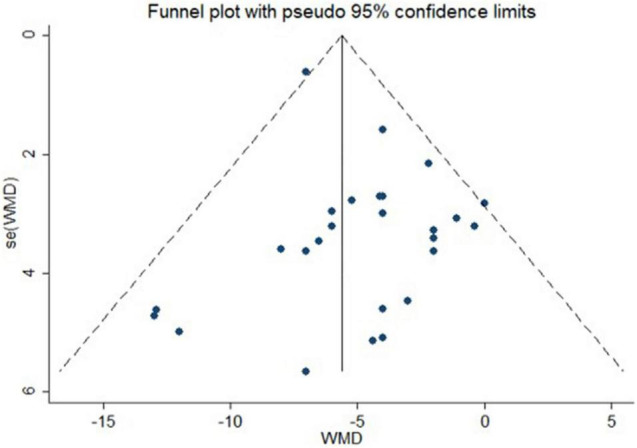
Funnel plot with pseudo 95% confidence limits for publication bias of actively controlled studies on central systolic blood pressure. WMD indicates weighted mean difference.

No publication bias was suggested by visual inspection of funnel plot in reporting changes in central SBP (Figure 6, the Egger’s test, *p* ≥ 0.08).

We also repeated analyses in subgroups according to the prespecified characteristics. In these subgroup analyses, the weighted mean differences were in agreement with the overall results (Supplementary Table 1).

When the trials of diuretics (*n* = 10) and β-blockers (*n* = 16) were compared, these older antihypertensive drugs behaved similar to the effects on the central SBP (*n* = 25) and peripheral SBP and PP (*n* = 24, *p* ≥ 0.17), but different to the effects on central PP (*n* = 19), central-to-peripheral PP amplification (*n* = 7), and central augmentation index (*n* = 24) in favor of β-blockers (*p* ≤ 0.002, Supplementary Table 2). Furthermore, when the trials of vasodilating (*n* = 4) and non-vasodilating β-blockers (*n* = 12) were compared, these two classes of β-blockers did not show significant difference in the effects on central and peripheral arterial hemodynamics (*p* ≥ 0.15, Supplementary Table 3).

## Discussion

This meta-analysis showed the differential effects of various antihypertensive drug classes on central hemodynamics. These results might help explain why some antihypertensive drugs, such as the β-blocker atenolol, were less efficacious in reducing the risk of stroke and cardiovascular mortality (Carlberg et al., 2004; McEniery, 2009), although it is generally believed that blood pressure reduction *per se* matters more than the choice of antihypertensive agents.

London et al. (1994) first conducted a controlled, blinded study to compare perindopril and nitrendipine in patients on chronic hemodialysis with a focus on central hemodynamic effects of vasoactive antihypertensive agents. The results showed that 12 months of treatment with ACEI and CCB had similar effects on augmentation index and carotid blood pressure. The following REASON study (Asmar et al., 2001) revealed in 471 hypertensive participants who were followed for 12 months that the combination of indapamide and perindopril decreased brachial SBP and PP more significantly than atenolol, with an adjusted between-group difference of −6.02 (95% CI, −8.90 to −3.14) and −5.57 mmHg (95% CI, −7.70 to −3.44), respectively. Similar adjusted between-group differences were observed for central SBP [−12.52 mmHg (95% CI, −17.97 to −7.08)] and PP [−10.34 mmHg (95% CI, −14.12 to −6.56)], and for carotid [−5.57% (95% CI, −10.77 to −0.36)] and aortic augmentation indices [−5.17% (95% CI, −7.74 to −2.61)].

A series of subsequent studies revealed a discrepancy in antihypertensive treatment on central and peripheral blood pressure. In a meta-analyses of 24 trials, Manisty and Hughes (2013) reported that treatment with β-blockers and diuretics posed a significantly less reduction in the central SBP than the brachial SBP by 6.9 and 6.8 mmHg, respectively, whereas other agents of monotherapy similarly lowered central and brachial SBP. Similar results were confirmed by McGaughey et al. (2016) in another meta-analyses of 52 studies with 4,381 participants and 58 studies with 3,716 participants for central SBP and augmentation index, respectively. Fifteen of the included studies had a crossover design, and 46 studies had a parallel group comparison design. Overall, antihypertensive drugs reduced brachial SBP more than central SBP by 2.52 mmHg, which was mainly attributed to the 5.19 mmHg greater reduction in the central-to-brachial amplification observed in β-blockers. Moreover, a significant reduction in the augmentation index was seen with RAS inhibitors, CCBs, and diuretics, but not β-blockers or α-blockers.

Both of the aforementioned meta-analyses were based on summary statistics instead of individual-subject data. The calculated differences in brachial and central SBP were less standardized in statistical analysis, such as adjustment for confounding factors. We tabulated head-to-head comparisons of various antihypertensive drugs regarding the effect on arterial hemodynamics. Despite similar reductions in peripheral PP, RAS inhibitors, and CCBs were more effective in reducing central PP than diuretics, β-blockers, and α-blockers. Newer antihypertensive agents significantly reduced more central blood pressure and augmentation than older agents.

The mechanisms for these differential treatment effects on central hemodynamics remain under investigation. As non-vasodilating β-blockers showed much less central blood pressure-lowering effect than the other classes of antihypertensive drugs, heart rate and vascular dilation or constriction must play a major role in the regulation of central hemodynamics. Indeed, in a meta-regression analysis (Ding et al., 2013), we previously found that slowing heart rate may to a large extent explain the less efficacy of β-blockers vs. the other classes of antihypertensive drugs. Although not shown in our present meta-analysis probably because of a limited number of trials, the vasoactive property must also play an important part in the central hemodynamic regulation. Indeed, a previous head-to-head comparison study showed divergent effects between vasodilating (nebivolol) and non-vasodilating (atenolol) β-blockers (Redón et al., 2014). Studies on the *I*_*f*_ inhibitor ivabradine provided further evidence. In a randomized, double-blind placebo-controlled, crossover study (Dillinger et al., 2015) in 12 patients with stable coronary artery disease, normal blood pressure, a sinus heart rate ≥70 beats per minute and β-blocker therapy, ivabradine treatment for 3 weeks reduced heart rate (−15.8 ± 7.7 vs. 0.3 ± 5.8 beats per minute, *p* = 0.001) and increased left ventricular ejection time (18.5 ± 17.8 vs. 2.8 ± 19.3 ms, *p* = 0.074) and diastolic perfusion time (215.6 ± 105.3 vs. −3.0 ± 55.8 ms, *p* = 0.0005), but did not significantly increase central SBP (−4.0 ± 9.6 vs. 2.4 ± 12.0 mmHg, *p* = 0.13) or augmentation index (−0.8% ± 10.0% vs. 0.3% ± 7.6%, *p* = 0.87). Taken together, it is probably the interaction between heart rate and vasoactive property that determines the extent of central pressure augmentation from wave reflections. This hypothesis may be tested in future animal experiments as well as human research. In addition, thiazide diuretics might be different in the central hemodynamic effects, for instance, between the so-called thiazide-type and thiazide-like diuretics. However, the present analysis did not allow us to perform this comparison because the thiazide-like diuretic was only used in one of the nine studies.

A major limitation of our meta-analysis was that two recent studies on an even newer antihypertensive drug class, i.e., angiotensin receptor neprilysin inhibitor (ARNI), were not included, because the comparative drug was an ARB (Schmieder et al., 2017; Williams et al., 2017), which was defined as a newer agent in the present analysis. In the PARAMETER (The Prospective comparison of Angiotensin Receptor neprilysin inhibitor with Angiotensin receptor blocker MEasuring arterial sTiffness in the eldERly) study, sacubitril/valsartan reduced central aortic systolic pressure (primary outcome) greater than olmesartan [between-treatment difference: −3.7 mmHg (95% CI, −6.4 to −0.9), *p* = 0.01] after 12 weeks of treatment but not after 52 weeks of treatment, probably because more subjects in the olmesartan group required add-on antihypertensive therapy than in the sacubitril/valsartan group (47% vs. 32%, *p* < 0.002). Indeed, Schmieder found that sacubitril/valsartan reduced central aortic PP to a greater extent than olmesartan (−3.5 mmHg, *p* = 0.01) after 52 weeks of treatment, with similar add-on treatment of amlodipine in the two groups (17.5% vs. 29.8%, *p* = 0.12). These observations shed some light on the potential beneficial effect of novel antihypertensive agents on central hemodynamics.

## Conclusion

Antihypertensive drug treatment with RAS inhibitors and CCBs was more efficacious than that with diuretics, β-blockers, and α-blockers in the central hemodynamic effects. At present, there is still no direct evidence regarding the clinical relevance of central hemodynamics for decision-making in the management of hypertension and cardiovascular prevention. Therefore, it is imperative to run adequately powered outcome trials to investigate whether central hemodynamic measurements are clinically useful in guiding antihypertensive treatment and other cardiovascular therapeutic approaches for the prevention of cardiovascular events.

## Data Availability Statement

The original contributions presented in the study are included in the article/Supplementary Material, further inquiries can be directed to the corresponding author/s.

## Author Contributions

J-GW and Y-BC conceived the study, performed the statistical analyses, and prepared the first draft of the manuscript. Y-BC and J-HX coordinated the data extraction. All authors participated in the interpretation of the data and approved the final version of the manuscript.

## Conflict of Interest

J-GW reports receiving lecture and consulting fees from Novartis, Omron, and Viatris. The remaining authors declare that the research was conducted in the absence of any commercial or financial relationships that could be construed as a potential conflict of interest.

## Publisher’s Note

All claims expressed in this article are solely those of the authors and do not necessarily represent those of their affiliated organizations, or those of the publisher, the editors and the reviewers. Any product that may be evaluated in this article, or claim that may be made by its manufacturer, is not guaranteed or endorsed by the publisher.
