# Supplementary material for: Antihypertensive Treatment and Central Arterial Hemodynamics: A Meta-Analysis of Randomized Controlled Trials
Source: Front Physiol. 2021 Nov 24;12:762586. doi: 10.3389/fphys.2021.762586 (PMC8652138; doi:10.3389/fphys.2021.762586)
Supplement: Supplementary file 1 [file Data_Sheet_1.doc]

**Online Supplemental Materials**

**Antihypertensive Treatments and Central Arterial Hemodynamics:
a Meta-analysis of Randomized Controlled Trials**

Running Title: Antihypertensive treatments and central hemodynamics

Yi-Bang Cheng, Jia-Hui Xia, Ji-Guang Wang.

**Correspondence:**

Ji-Guang Wang, MD, PhD

The Shanghai Institute of Hypertension

Ruijin 2nd Road 197,

Shanghai 200025, China

Phone: +86-21-64370045-610911

Fax: +86-21-64662193

Email:jiguangwang@aim.com

**Table of Contents**

**Table S1** Sensitivity analysis of the weighted mean difference in central systolic blood pressure and augmentation index according to several prespecified characteristics of trials

**Table S2** The weighted mean differences in central and peripheral hemodynamics for diuretics or β-blockers versus newer antihypertensive drugs

**Table S3** The weighted mean differences in central and peripheral hemodynamics for vasodilating or nonvasodilating β-blockers versus newer antihypertensive drugs

**Figure S1** Effects of renin-angiotensin-aldosterone system inhibitors and calcium-channel blockers versus diuretics, β-blockers, and α-blockers on peripheral systolic blood pressure and pulse pressure

**Table S1**

**. Sensitivity analyses of the weighted mean differences in central systolic blood pressure and augmentation index according to several prespecified characteristics of trials**

|  |  | **Central Systolic Blood Pressure** | | | |  | **Central Augmentation Index (%)** | | | |
| --- | --- | --- | --- | --- | --- | --- | --- | --- | --- | --- |
|  |  | Nº of arms | Nº of patients | WMD (95% CI, mmHg) | *P* |  | Nº of arms | Nº of patients | WMD (95% CI, %) | *P* |
| Jadad scores ≥ 3 |  | 19 | 2240 | -4.22 (-5.64 to -2.79) | <0.001 |  | 17 | 1673 | -6.90 (-9.26 to -4.55) | <0.001 |
| SphygmoCor radial approach |  | 17 | 1420 | -4.07 (-5.55 to -2.60) | <0.001 |  | 17 | 1420 | -5.57 (-7.76 to -3.38) | <0.001 |
| Diagnosis of hypertension |  | 21 | 2369 | -5.59 (-6.47 to -4.70) | <0.001 |  | 22 | 1881 | -5.50 (-7.34 to -3.66) | <0.001 |

The weighted mean differences (WMD) and confidence intervals (CI) were computed for active antihypertensive treatment versus placebo or no-treatment or for newer versus older antihypertensive drugs. Weights are from the fixed- and random-effects models for central systolic blood pressure and augmentation index, respectively.

**Table S2**

**.** **The weighted mean differences in central and peripheral hemodynamics for diuretics or β-blockers versus newer antihypertensive drugs**

|  |  | **Diuretics** | | |  | **β-Blockers** | | | *P* for heterogeneity between groups |
| --- | --- | --- | --- | --- | --- | --- | --- | --- | --- |
|  |  | Nº of arms | Nº of patients | WMD (95% CI, mmHg) |  | Nº of arms | Nº of patients | WMD (95% CI, %) |
| Central |  |  |  |  |  |  |  |  |  |
| Systolic blood pressure (mm Hg) |  | 10 | 1122 | -6.24 (-7.31 to -5.18) |  | 15 | 1402 | -4.25 (-5.80 to -2.70) | 0.17 |
| Pulse pressure (mm Hg) |  | 7 | 781 | -1.27 (-3.25 to 0.72) |  | 12 | 1075 | -4.55 (-6.85 to -2.45) | 0.002 |
| Peripheral |  |  |  |  |  |  |  |  |  |
| Systolic blood pressure (mm Hg) |  | 10 | 1122 | -2.27 (-3.84 to -0.70) |  | 14 | 1314 | -1.52 (-2.89 to -0.14) | 0.32 |
| Pulse pressure (mm Hg) |  | 6 | 918 | -0.76 (-2.38 to 0.87) |  | 10 | 805 | -0.99 (-2.31 to 0.33) | 0.83 |
| Central-to-peripheral pulse pressure amplification (mm Hg) |  | 3 | 131 | 0.01 (-0.07 to 0.10) |  | 4 | 612 | 0.21 (0.13 to 0.29) | 0.001 |
| Central augmentation index (%) |  | 9 | 643 | -4.60 (-7.23 to -1.48) |  | 15 | 1393 | -7.37 (-9.50 to -5.23) | <0.001 |

The weighted mean differences (WMD) and confidence intervals (CI) were computed for diuretics or β-blocker versus placebo, no-treatment or newer antihypertensive drugs. Weights are from the fixed-effects models for central and peripheral systolic blood pressure, peripheral pulse pressure, and central-to-peripheral pulse pressure amplification, and from the random-effects models for central pulse pressure and augmentation index.

**Table S3**

**. The weighted mean differences in central and peripheral hemodynamics for vasodilating or nonvasodilating β-blockers versus newer antihypertensive drugs**

|  |  | **Vasodilating β-blockers** | | |  | **Nonvasodilating β-blockers** | | | *P* for heterogeneity between groups |
| --- | --- | --- | --- | --- | --- | --- | --- | --- | --- |
|  |  | Nº of arms | Nº of patients | WMD (95% CI, mmHg) |  | Nº of arms | Nº of patients | WMD (95% CI, %) |
| Central |  |  |  |  |  |  |  |  |  |
| Systolic blood pressure (mm Hg) |  | 4 | 319 | -2.69 (-5.31 to -0.06) |  | 11 | 1083 | -5.08 (-7.00 to -3.16) | 0.15 |
| Pulse pressure (mm Hg) |  | 4 | 319 | -3.03 (-4.92 to -1.13) |  | 8 | 756 | -6.32 (-10.17 to -2.47) | 0.47 |
| Peripheral |  |  |  |  |  |  |  |  |  |
| Systolic blood pressure (mm Hg) |  | 4 | 319 | -1.41 (-3.94 to 1.11) |  | 10 | 927 | -1.56 (-3.20 to 0.08) | 0.92 |
| Pulse pressure (mm Hg) |  | 3 | 137 | 0.57 (-2.03 to 3.18) |  | 7 | 668 | -1.52 (-3.05 to 0.002) | 0.17 |
| Central-to-peripheral pulse pressure amplification (mm Hg) |  | 4 | 612 | 0.21 (0.13 to 0.29) |  | - | - | - | - |
| Central augmentation index (%) |  | 4 | 319 | -7.68 (-10.56 to -4.81) |  | 11 | 1074 | -7.52 (-10.24 to -4.80) | 0.85 |

Vasodilating β-blockers included nebivolol and carvedilol. Nonvasodilating β-blockers included atenolol, bisoprolol and labetalol. The weighted mean differences (WMD) and confidence intervals (CI) were computed for diuretics or β-blocker versus placebo, no-treatment or newer antihypertensive drugs. Weights are from the fixed-effects models for central and peripheral systolic blood pressure, peripheral pulse pressure, and central-to-peripheral pulse pressure amplification, and from the random-effects models for central pulse pressure and augmentation index.

**Figure S1**

**.** **Effects of renin-angiotensin-aldosterone system inhibitors and calcium-channel blockers versus diuretics, β-blockers, and α-blockers on peripheral systolic blood pressure and pulse pressure**

**
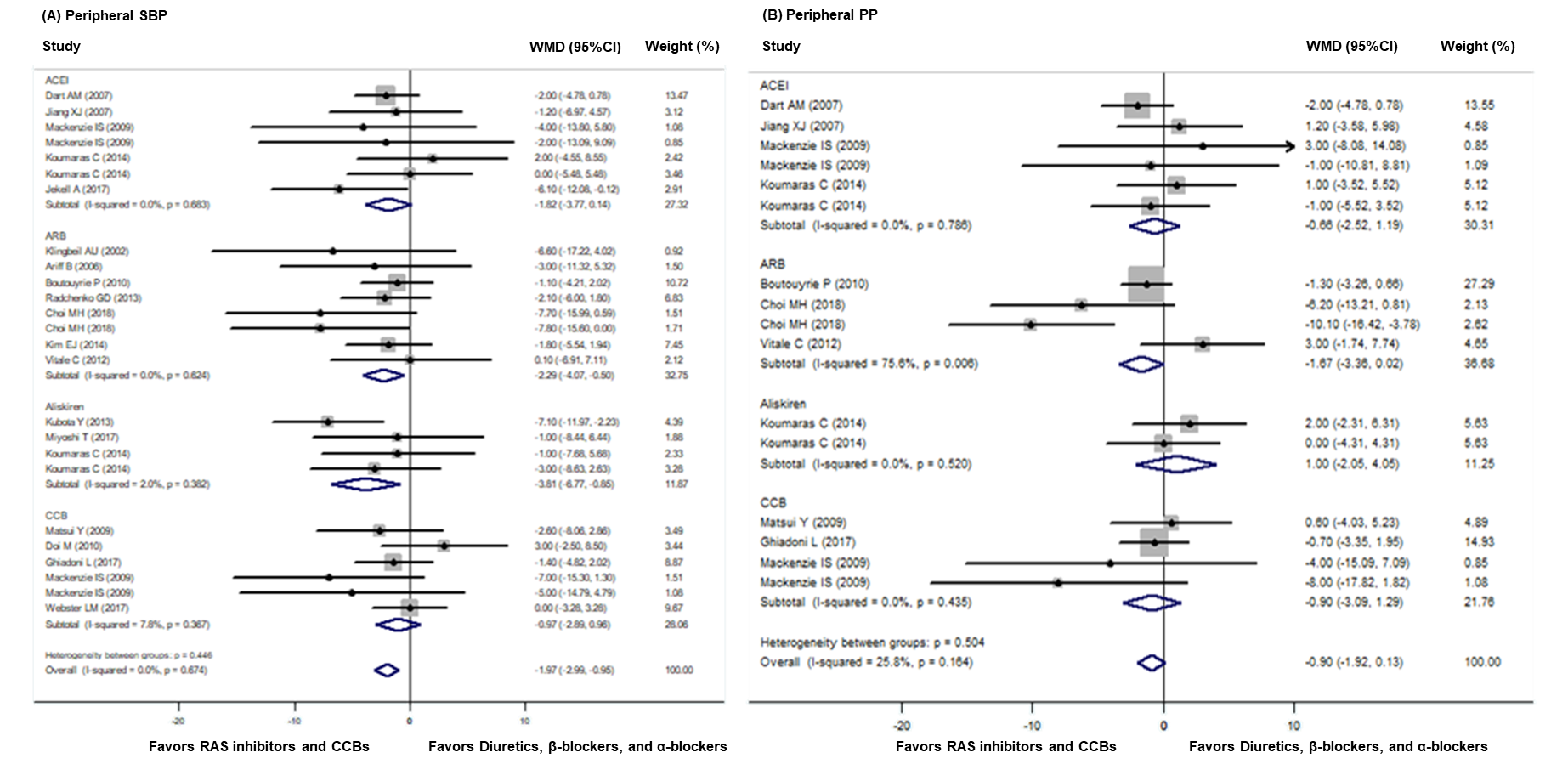
**

Dots represent mean difference of each study. The size of the squares is proportional to the sample size. Horizontal lines represent the 95% confidence intervals. Open diamonds represent the weighted mean difference (WMD) with 95% confidence intervals (CI). Weights are from the random effects analysis.
